# Airway disease phenotypes in animal models of cystic fibrosis

**DOI:** 10.1186/s12931-018-0750-y

**Published:** 2018-04-02

**Authors:** Alexandra McCarron, Martin Donnelley, David Parsons

**Affiliations:** 1 Adelaide Medical School, Discipline of Paediatrics, University of Adelaide, Adelaide, SA Australia; 2 Department of Respiratory and Sleep Medicine, Women’s and Children’s Hospital, Adelaide, SA Australia; 3 Robinson Research Institute, University of Adelaide, Adelaide, SA Australia

**Keywords:** Cystic fibrosis, Animal models, Lung disease, Pathology

## Abstract

In humans, cystic fibrosis (CF) lung disease is characterised by chronic infection, inflammation, airway remodelling, and mucus obstruction. A lack of pulmonary manifestations in CF mouse models has hindered investigations of airway disease pathogenesis, as well as the development and testing of potential therapeutics. However, recently generated CF animal models including rat, ferret and pig models demonstrate a range of well characterised lung disease phenotypes with varying degrees of severity. This review discusses the airway phenotypes of currently available CF animal models and presents potential applications of each model in airway-related CF research.

## Background

Animal models of cystic fibrosis (CF) are crucial for understanding CF pathogenesis and developing therapeutic strategies. Given that CF affects multiple organs including the gastrointestinal tract, lungs, pancreas, liver, and reproductive organs, it desirable to generate animal models that accurately capture all disease facets. For the past 20 years, in vivo CF-related research has predominately used genetically modified murine models [1]. These mouse models have proven invaluable for studying aspects of CF pathophysiology, however, they have important limitations. Obvious disparities between murine and human anatomy and physiology mean that phenotypes observed in humans are not always reproduced in mice [2]. Investigating some features of CF airway disease in mouse models has proven problematic, as CF mice fail to develop hallmark features including mucus obstruction, chronic bacterial infections, and persistent inflammation. Of all clinical manifestations associated with human CF, progressive lung disease is the predominant cause of morbidity and mortality among patients [3]. As such, an accurate animal model of CF lung disease initiation and progression is imperative for investigating pathogenesis, identifying potential treatment targets, and testing experimental therapies.

Following the generation of CF murine models, it became apparent that species characteristics are important to consider when developing an animal model of CF lung disease. Traits that may influence the choice of species include airway cellular architecture, particularly the distribution and abundance of submucosal glands, dominance of alternative chloride secretory pathways, and conservation of the structure and function of the cystic fibrosis transmembrane conductance regulator (CFTR) protein [1]. Since the development of CF mouse models, advances in genetic engineering have facilitated the generation of a number of alternative models including a CFTR knockout rat [4], knockout ferret [5], two knockout pig models [6, 7], and a pig harbouring the common Phe508del CFTR mutation [6]. There are also early reports of CFTR knockout and Phe508del mutant CF rabbit models being developed [8]. Due to the similarities between the ovine and human lung, generation of a CF sheep model has long been proposed [9]. Although a CF sheep model has not yet been established, rapid advances in gene editing could promote its generation in the near-future.

This review details the airway disease phenotypes of existing CF animal models, and examines their strengths and weaknesses for basic and pre-clinical research.

### CF lung disease pathophysiology

Understanding the underlying pathophysiology of CF lung disease is necessary prior to examining the phenotypes observed in animal models. Under normal conditions, the CFTR protein functions as an epithelial anion channel responsible for cyclic-AMP-dependent chloride and bicarbonate secretion, as well as the regulation of epithelial sodium channels (ENaC) [10]. CF arises when mutations occur in the CFTR gene, subsequently disrupting epithelial ion transport. To date, over 2000 disease-causing CFTR mutations have been identified [11]. These mutations are classified into 6 categories based on the mechanism of CFTR dysfunction including defective protein synthesis and processing, dysfunctional channel regulation and conduction, and reduced protein synthesis and stability [12]. The most common CF-causing mutation is the Class II mutation Phe508del, with approximately 90% of CF patients carrying one copy [11].

Although the role of CFTR in transepithelial ion transport is widely accepted, the exact mechanisms underlying CF lung disease development have long been debated. Several hypotheses have been proposed regarding the pathogenesis of CF lung disease [13]. The leading theory with a large body of supporting evidence is known as the “low volume” hypothesis [14–16]. This theory postulates that reduced transepithelial chloride transport due to dysfunctional CFTR, as well as increased sodium absorption caused by a lack of CFTR-dependent inhibition of ENaC, creates an ion imbalance that leads to osmotically-driven water absorption into the tissues, and in turn, reduced airway surface layer (ASL) hydration [3, 10]. The depleted ASL results in dehydration of the mucus layer and consequently, viscous mucus adheres to airway surfaces. Onset of mucus-stasis and impaired mucociliary clearance (MCC) result in ineffective removal of inhaled microorganisms [17]. Bacteria adhere to the airway mucus eventually resulting in the formation of biofilms that effectively evade antimicrobial substances and host neutrophils. Over time, more resistant and atypical organisms colonise the airways including mycobacteria, yeast, and fungus. A state of chronic lung infection incites persistent inflammatory responses that lead to destruction of the airway tissue, bronchiectasis, progressive loss of lung function, and ultimately respiratory failure [18].

### Murine models

Shortly after the discovery of the CFTR gene in 1989 the first CF mouse model was generated [19]. Since then, at least 15 CF mouse models have been developed and characterised (reviewed in detail elsewhere [20–22]). Two major groups of CF mouse models exist: null alleles (knockouts) with no detectable mRNA or functional CFTR protein, and mutant alleles with common human CF-causing mutations (e.g. Phe508del or Gly551Asp) introduced into the mouse CFTR sequence [23]. The limited utility of CFTR knockout strains due to early-fatal intestinal obstruction led to the development of a third category; “gut corrected” transgenic CF mice. In this model, human CFTR cDNA is expressed under the control of the rat fatty acid binding protein (FABP) promoter, thereby localising expression to the intestinal epithelium [24], and subsequently increasing the longevity of CF mice [25]. Humanised mouse models that exclusively express human CFTR with common CF-causing mutations are also currently under development [26].

#### Lung disease phenotype in CF mice

Disease phenotypes and severity among CF mouse models tend to be heterogeneous due to the diversity of genetic backgrounds and variation in gene targeting strategies used to generate the animals [25]. Although early characterisation studies initially reported that CF mice did not demonstrate any lung pathology [19], emerging evidence suggests that they do in fact exhibit some phenotypes reminiscent of human CF lung disease. Similar to the early pulmonary phenotype observed in human CF airways, multiple studies have demonstrated that CF mice display altered respiratory mechanics [27–30]. Furthermore, when a state of hyperinflammation is induced in the lungs of CF and wild-type mice using repeated exposure of an inflammatory stimuli (in this case lipopolysaccharide from *Pseudomonas aeruginosa*), CF mouse lungs undergo remodelling and morphological changes, while the airways of wild-type mice are able to efficiently recover [31]. Recent studies also indicate that CF mice spontaneously acquire *Bordetella* respiratory tract infections [32].

Although CF mice demonstrate features of lung disease, their use is somewhat limited as they do not exhibit the severe pathology characteristic of established human CF lung disease consisting of chronic respiratory infection, inflammation, mucus plugging, and progressive bronchiectasis [23]. Several theories may explain why CF mice fail to develop the overt lung disease that is observed in humans. One is that modifier genes are upregulated in CF mice, leading to partial correction of the underlying ion transport defects. Dominance of the calcium-activated chloride channel (CACC) secretory pathway has been observed in the airway epithelia of CF mice [33]. It has been suggested that this adaptive mechanism compensates for defective CFTR-mediated chloride transport, thereby rectifying the underlying ion imbalance, and protecting the murine airways from disease [33, 34]. In addition to upregulation of the CACC in CF murine airways, the cAMP-mediated CFTR pathway normally has a less dominant role in the respiratory epithelium of mice [25].

The lower airways of adult CF mice do not demonstrate electrical defects that are characteristic of human CF airways, including reduced cAMP-mediated chloride secretion and sodium hyperabsorption. Hyperactivity of ENaC is important in the pathogenesis of CF lung disease as it is thought to contribute to the depletion of the ASL and subsequent cascade of events including mucus accumulation, impaired MCC, infection and inflammation, and lung tissue damage [35]. As such, a lack of sodium hyperabsorption in the lower airways of CF murine models may explain the absence of lung disease development [25]. It is also possible that the structure and function of CFTR evolved differently in mice. Investigations into CFTR homology among species reveal that mouse CFTR is only 88% conserved with human CFTR at the amino acid level, as such, the murine CFTR channel exhibits some different pharmacological and gating properties to human CFTR [36, 37].

CFTR protein in human airways is predominately expressed in the ciliated epithelium and the submucosal glands, therefore histological disparities between murine and human airways may explain the absence of lung disease in CF mice [38, 39]. Mouse and human airways differ in cellular architecture; human distal airways are comprised primarily of ciliated cells, whereas murine lower airways are largely non-ciliated, secretory, club cells [25]. Furthermore, human airways have numerous submucosal glands throughout the trachea and bronchi, while murine airways only have a small proportion of these glands in the larynx and proximal trachea [40]. As submucosal glands are implicated in human CF airway disease, their scarcity in the murine distal airways could contribute to the lack of lung pathophysiology [41].

Environmental factors and host-pathogen interactions may also play a role [20]. It has been hypothesised that CF lung disease is not effectively modelled without the presence of pathogens, therefore the conventional housing of CF mice in semi-sterile, and often specific pathogen free (SPF) conditions may prevent development of the lung disease that is typically observed in humans with CF [22]. However, this theory does not appear to be substantiated, as CF mice reared in a non-sterile environment still fail to develop lung disease [25].

#### Upper airway and tracheal phenotype in CF mice

Some CF mouse strains display a mild phenotype in the upper airways and trachea. Characteristics observed include distended submucosal glands in the nasal mucosa [42], atrophy of serous gland tissue in the sinuses [19], hyperplasia of goblet cells in the nasal septa, and a reduced ASL in the nasal epithelium [15]. CFTR^−/−^ mouse models also show notable tracheal abnormalities such as incomplete cartilage rings [43], and reduced smooth muscle area [44]. Impaired MCC in the trachea of CFTR^tm1HGU^ and CFTR^tm1UNC^ strains has also been reported [45], however, these findings have been contested by others that found MCC was unaffected in CFTR^tm1UNC^ mice [46].

The nasal epithelium of CF mice demonstrates similar bioelectric abnormalities to human CF airways including reduced chloride transport and sodium hyperabsorption. These defects have been well documented using the nasal potential difference (NPD) measurement technique; an assessment that involves placing fluid-filled cannula electrodes (connected to a millivoltmeter) on the nasal lining to measure the electrical potential produced by ion transport across the epithelium in response to different salt solutions [47]. All CF mice appear to exhibit reduced cAMP-mediated chloride secretion in the nasal epithelium, which is characteristic of human CF airways [22, 25]. The hyperactivity of ENaC, a feature present in human CF nasal epithelium [48], is also observed in the nasal epithelium of most CF mouse models including knockout [49], CFTR^∆F508^ [49, 50] and CFTR^G551D^ strains [51]. When amiloride (a drug that blocks ENaC-mediated sodium absorption) is perfused through the nasal epithelium of a CF mouse, a significant depolarisation response occurs, which is consistent with the presence of sodium hyperabsorption [21].

As the nasal epithelium of CF mice accurately recapitulates the electrophysiological profile of human CF airways, it has proven valuable for testing therapeutic agents that replace dysfunctional CFTR (i.e. gene therapies) [52], and those that restore normal ion transport [53, 54]. Mouse strains bearing the Phe508del and Gly551Asp mutations could be particularly useful for testing compounds that correct CFTR processing and trafficking (CFTR correctors), and defective channel gating (CFTR potentiators). However, some CFTR potentiators that strongly augment human CFTR do not have any effect on murine CFTR, most likely due to differences in the murine CFTR channel properties [20]. Once developed, hCFTR CF mouse models will be valuable for trialling small molecule drugs. Although the nasal epithelium of CF mice has provided a useful platform for trialling therapeutics [52–54], it should be noted that murine nasal mucosa is composed of 40% olfactory epithelium and 60% respiratory epithelium (depending on location), thus differing substantially from the cellular composition of human lungs, the intended target organ of these therapies [12].

#### Bacterial challenge rodent models

The absence of overt lung disease in CF mouse models, even under non-sterile conditions, led some investigators to trial more radical bacterial-challenge approaches to simulate a chronically infected and inflamed airway [20]. In these studies, a range of knockout and mutant CF strains were inoculated with *P. aeruginosa*, an important pathogen in the development and progression of human CF lung disease [55]. Bacterial challenge with *P. aeruginosa* has previously yielded variable results, most likely due to differences in dose, delivery method, dosing frequency, and the clinical isolate used. Despite this, many of the challenged CF mouse strains demonstrated susceptibility to acute *P. aeruginosa*, characterised by increased mortality and reduced ability to clear the bacteria when compared to non-CF control animals [55–58].

In one example, *P. aeruginosa* laden agarose beads were intratracheally instilled into CFTR^tm1UNC^ mice. In addition to increased mortality (when compared to non-CF controls), challenged mice also demonstrated a significant pulmonary inflammatory response following inoculation, indicated by elevated levels of inflammatory markers in bronchoalveolar lavage (BAL) [55]. Studies have also attempted to deliver *P. aeruginosa* to CF mice in drinking water, resulting in low levels of chronic colonisation within CF airways, while wild-type animals effectively cleared the bacteria [59]. Other CF-related bacterial strains have also been used, in one study CFTR^tm1UNC^ mice were intranasally challenged with the bacterium *Burkholderia cepacia* (*B. cepacia)*. Following instillation, bacteria persisted in the airways of CF mice and caused severe bronchopneumonia while wild-type mice remained healthy [60].

These bacterial challenge studies indicate that the absence of CFTR in mouse airways is enough to produce heightened susceptibility to CF-related pathogens [58]. Accordingly, challenged CF mice elicit a sustained inflammatory response that could allow for investigations into the relationship between inflammation and infection in a CFTR-deficient lung [57], as well as trialling of novel anti-inflammatory and antibiotic therapies [60, 61]. However, these bacterial challenge mouse models are limited, as they do not reflect the conditions in which CF patients acquire and respond to infection. Furthermore, instilling CF mice with human pathogens is a somewhat naïve approach as host-pathogen interactions are complex, differ in mice and humans, and do not solely depend on CFTR activity [20].

In addition to bacterial challenge being performed in CF mice, models of respiratory infection have also been established in normal rodents by using key CF-related bacterial species. Mouse models of chronic *Burkholderia cenocepacia* [62] and *P. aeruginosa* [63] infection have recently proven useful for investigating host-pathogen interactions of bacterial species that are pertinent to CF lung disease. Similarly, a rat model of chronic *P. aeruginosa* infection that exhibits pathologic features of human *P. aeruginosa* pulmonary infection has also been developed [64].

#### Congenic CFTR knockout mouse model

It has been hypothesised that generation of CF mouse models on mixed genetic backgrounds results in upregulation of modifier genes that prevent the development of lung disease. To test this theory, the CFTR^tm1UNC^ CF mouse strain was redeveloped on a single genetic background and was termed B6-CFTR^tm1UNC^. Interestingly, B6-CFTR^tm1UNC^ mice develop lung disease manifestations including bronchiolar mucus retention, tissue fibrosis, hyperinflated alveoli, and alveolar wall thickening [65]. B6-CFTR^tm1UNC^ mouse lungs also show inflammatory cell recruitment with an influx of neutrophils observed. This inflammatory disease phenotype appears spontaneous, as analysed mice were housed in SPF conditions with no airway pathogens detected prior to, or during the onset of inflammation [66].

In the nose, PD measures indicate that the congenic CF mouse demonstrates reduced chloride secretion, which is consistent with an absence of CFTR. Unlike mixed-background CF mouse strains however, the response to amiloride does not appear to differ between congenic CF mice and wild-type, suggesting that congenic CF mice do not exhibit sodium hyperabsorption in the nasal epithelium [65]. Furthermore, B6-CFTR^tm1UNC^ mice challenged with *P. aeruginosa* demonstrate reduced capacity to control infection [67]. It has been speculated that congenic B6-CFTR^tm1UNC^ mice develop aspects of lung disease as they lack, or fail to activate alternate chloride conductance pathways normally present in mixed background CF mouse models [65].

The B6-CFTR^tm1UNC^ mouse is one of the few models that exhibits inflammatory lung disease, as such it has proven useful for exploring the long-debated question of whether inflammation in CF airways is spontaneous, or induced by infection [66]. Even though the B6-CFTR^tm1UNC^ mouse demonstrates aspects of CF lung disease, the original mixed-background CF mouse strains continued to be used. Unexpectedly, generation of another congenic mouse model based on the CFTR^tm1HGU^ strain resulted in amelioration of the CF phenotype, rather than an increase in severity [68]. Given the potential undesired effects of developing models on mixed-backgrounds, along with the rapid uptake of new gene editing technologies such as CRISPR/Cas9, newly generated CF mouse models are likely to be congenic.

#### β-ENaC mouse model

Difficulties recapitulating the pathophysiology of CF lung disease in existing mouse models led to the development of the β-ENaC mouse (also known as the *Scnn1b* mouse). The β-ENaC mouse is a transgenic mouse model that overexpresses the β-subunit of ENaC in the lungs, thereby mimicking the sodium ion transport abnormalities observed in CF human airways [69, 70]. The airways of β-ENaC mice recapitulate the key processes of CF lung disease initiation, whereby increased airway sodium absorption results in depletion of the ASL and deficient mucus clearance. Accordingly, β-ENaC mice exhibit CF-like lung disease with features including mucus hypersecretion, mucus obstruction in the conducting airways, MCC impairment (as measured directly by microdialysis), goblet cell metaplasia, and neutrophilic airway inflammation [69–71].

Initial studies performed using adult β-ENaC mice failed to detect spontaneous bacterial infection in the airways, however, when intratracheally challenged with *Haemophilus influenzae* and *P. aeruginosa,* adult β-ENaC mice exhibit impaired pathogen clearance when compared to wild-type mice [69]. More recent studies involving longitudinal analysis of BAL indicate the presence of spontaneous lung infection in neonatal β-ENaC mice, while wild-type neonates had no culturable bacteria. The same study also revealed that the lung bacterial burden and proportion of infected β-ENaC mice appears to decrease with age, attributable to maturation of the immune system that occurs during the postnatal period [72].

The β-ENaC mouse provides a relevant model for investigating CF lung disease pathogenesis, particularly the interactions between ion transport, the ASL, and MCC. The lung disease modelled by the β-ENaC mouse has already proven useful for developing novel respiratory diagnostic tools to localise and measure heterogeneity of lung disease [73]. Furthermore, β-ENaC mice could be used to evaluate a range of treatments including those that target ASL depletion, mucus obstruction, and inflammation [70]. For instance, the β-ENaC mouse could be used to trial ENaC inhibition strategies that may restore normal sodium transport. Such approaches include use of ENaC antagonists, silencing ENaC expression using short-interfering RNAs (siRNAs), and inhibiting proteases that activate ENaC [35]. However, as CFTR expression and function are not altered in the β-ENaC model, it is not suitable for testing therapeutics that replace, correct or potentiate CFTR [70].

### CF rat model

Limited usability of CF mouse models attracted researchers to other species to use as platforms for developing a more accurate CF model. Compared to larger animals, rats have the advantages of a short gestation (21–23 days) and early sexual maturity (8 weeks), thereby allowing for rapid breeding. Rats also have fewer animal husbandry expenses compared to large animals, and have been used widely for research purposes meaning they are well characterised in terms of physiology, pharmacology, and toxicology [74]. Moreover, there is a vast array of rat-specific molecular tools available that would be difficult to source or develop for other species. Unlike mice, rats have extensive submucosal glands present throughout the cartilaginous airways (trachea and primary bronchi), similar to humans [75]. As submucosal glands are implicated in the development of CF lung disease in humans [76], rat lungs are an attractive model.

#### Airway disease in the CFTR knockout rat model

CFTR^−/−^ rats recapitulate important features of lung disease that are observed in humans with CF. Histologically, the respiratory epithelium of the nasal septum in CF rats is normally developed, however, cells tend to exhibit dilation due to increased levels of intracellular mucus, indicative of defective mucus secretion. Morphologically, the trachea of ≤ 6-week-old CF rats appears to develop abnormally, with evidence of diminished tracheal cartilage and gland area when compared to wild -type rats. CF rats also display significant depletion of the ASL with a notable reduction in periciliary liquid (PCL) depth [4]. Furthermore, preliminary findings indicate that young CF rats demonstrate a hyperacidic airway surface pH [77], similar to that observed in CF pigs [78]. Interestingly, a recent study has revealed that ASL pH is not reduced in children with CF [79].

Despite depletion of the ASL, mucociliary transport (MCT) appears to be unaffected in young CF rats (< 3 months) [4]. However, early evidence suggests that as the animal ages and the airway submucosal glands develop, MCT rates reduce significantly when compared to wild-type. Preliminary investigations also suggest that by 6 months of age, CF rats exhibit mucus plugging of submucosal glands in the large airways [77, 80]. Spontaneous infection and inflammation is not present in CF rat airways, with no differences observed in BAL profiles between wild-type and CFTR^−/−^ genotypes [4]. Although spontaneous infection is absent, emerging evidence indicates that similar to CF mice, CF rats have a diminished ability to clear induced *P. aeruginosa* infection [81].

CF-like electrophysiological defects are present in the both the nasal and tracheal epithelium of CF rats as measured by NPD and short circuit current (I_sc_), respectively. In the nasal epithelium, CF rats demonstrate reduced chloride transport and show no evidence of cAMP-mediated chloride secretion following attempts to stimulate CFTR with forskolin (a cAMP agonist), both features consistent with an absence of CFTR. I_sc_ measures of transepithelial ion transport performed on freshly excised CF rat tracheal tissue using Ussing chambers demonstrates a markedly lower baseline I_sc_ in CF rats when compared to wild-type, typical of a CF bioelectric profile. Furthermore, administration of an inhibitor of CFTR-dependent chloride transport to the tracheal tissue has minimal effect on I_sc_, suggesting low basal CFTR activity [4]. CFTR^−/−^ and wild-type rats demonstrate similar responses to amiloride in both the nasal and tracheal epithelium, suggesting that unlike human CF airways and the nose of CF mice, CF rat airways do not demonstrate sodium hyperabsorption.

Recapitulation of key CF airway features in the CFTR^−/−^ rat model including reduced ASL and MCT will provide opportunities to further investigate the mechanisms involved in lung disease development. Electrophysiological, MCT and ASL measures may also be useful in assessing the efficacy of airway-targeted treatments including genetic therapies. Despite these and many other potential applications, the advantages of CF rats such as their small size, rapid breeding, ease of husbandry, and display of key airway disease phenotypes, are yet to be exploited [4]. Additional in-depth investigations of the airway phenotype are required to reinforce initial characterisation reports, and longitudinal studies are necessary to follow lung disease development over the lifetime of the animals. Furthermore, carrying out bacterial challenge studies in CF rat airways like those performed in CF mice [57], may reveal disease characteristics that are otherwise undetected in a non-infected lung. Recent advances in gene editing will also enable the future development of CF rat strains carrying human-specific CFTR mutations.

### CF ferret model

Due to similarities between ferret and human lung cell biology and anatomy, normal ferret lungs have been used to model human pulmonary infections including severe acute respiratory syndrome [82], and influenza virus [83]. Ferret airways also have important features that make them attractive for developing a model of CF lung disease. Like humans, ferret airways have submucosal glands throughout the trachea and primary bronchi that express high levels of CFTR [38, 84]. Additionally, the predominant secretory cell type in ferret and human proximal cartilaginous airways is the goblet cell, unlike in mice, where the equivalent is the club cell [85]. Ferret CFTR also has a high degree of amino acid sequence conservation with human CFTR (95%), and accordingly, the bioelectric and pharmacologic properties of CFTR are similar in ferrets and humans [36].

#### Airway disease in the CFTR knockout ferret model

The CF ferret demonstrates a severe lung phenotype that is heterogeneous between individual animals. Mucus plugging is observed in the small and large airways, and in some cases, causes complete blockage. In addition to the presence of mucus, pockets of trapped air and deflated alveoli (atelectasis) are also present. Dilation of the submucosal glands and ducts, goblet cell hyperplasia, and presence of inflammatory cells and bacterial colonies within the mucus are all observed in CF ferret airways. In instances where bronchopneumonia is present, purulent inflammation, necrosis, and pulmonary consolidation can also occur. CF ferrets also occasionally present with mucus accumulation and inflammation in the sinuses. When MCC is measured using fluorescent bead migration in ex vivo tracheas, CF ferrets exhibit a significantly reduced (by seven-fold) MCC rate in comparison to their non-CF counterparts [86].

Bioelectric I_sc_ measures performed on CF ferret tracheal tissue demonstrate electrophysiological abnormalities that are typical of CF [5]. CF ferret tracheal epithelia exhibits a large reduction in transepithelial current when an inhibitor of non-CFTR epithelial chloride channels is delivered, suggesting decreased CFTR activity. Furthermore, delivery of various cAMP agonists (e.g. forskolin) to the tracheal tissue does not appear to stimulate significant responses, and subsequent addition of a CFTR inhibitor also results in little change in ion transport, features that are all consistent with a lack of CFTR function and subsequent defective cAMP-dependent chloride transport [87]. Fluid secretion from the submucosal glands is also substantially reduced in CF ferret tracheal tissue, a feature that is also present in human CF proximal airways [5]. Interestingly, there is no difference in ENaC activity in tracheal tissue taken from CF and wild-type animals > 3 months of age (as measured by Ussing chamber I_sc_ analysis). However, there is some evidence that CF ferrets display an age-dependent increase in ENaC activity that is not observed in wild-type controls, suggesting a possible link between ENaC activity and airway disease progression [86].

In striking contrast to CF rodent models, CF ferrets are highly susceptible to lung infection, and antibiotic treatment is required from birth to ensure survival. Bacteriologic studies have identified a diverse range of bacteria in the lungs of CF ferrets with the most common species from the genera *Streptococcus, Staphylococcus,* and *Enterococcus.* Interestingly, enteric bacterial species from the *Enterococcus* and *Escherichia* genera are abundant in the lungs of CF ferrets. The significant overlap in lung and intestinal flora suggests that the intestines provide a primary source of lung-colonising bacteria [5].

The CF ferret is one of the few models that exhibits spontaneous lung infection, a crucial factor implicated in CF lung disease. However, the onset of lung infection in the CF ferret is rapid and severe, differing from the slow progressing chronic infection observed in humans [88]. As CF ferrets are susceptible to lung infections continuous antibiotic treatment and ongoing high levels of care are required from birth to improve survival [5]. Along with infection, the ferret lungs demonstrate mucus plugging, inflammation, and reduced MCC, all important manifestations of a CF airway. Given this, the ferret could be useful for testing the efficacy of potential therapeutics under conditions of infection and inflammation, which has not been possible with previous CF models. The presence of spontaneous lung infection will also assist in understanding the complex changes in the CF lung microbiome with disease progression [86]. One disadvantage of the CF ferret model is the lack of ENaC dysregulation in the airway tissues, a process thought to be significant in the pathogenesis of lung disease [36].

Along with lung disease, CF ferrets also develop a range of gastrointestinal pathologies, similar to those observed in CF patients. Notably, CF ferrets demonstrate a high prevalence of meconium ileus at birth (75% of kits) that is often fatal, as well as pancreatic dysfunction, malnutrition, and liver disease [5, 89]. To alleviate this severe gastrointestinal phenotype, a CFTR^−/−^ model expressing ferret CFTR cDNA under direction of the FABP promoter has been developed. This gut-corrected model will enable more CF ferrets to survive to maturity thereby allowing lung disease progression to be investigated longitudinally [5].

### CF pig models

Parallels between humans and pigs in regards to anatomy, physiology, biochemistry, size, life span, and genetics make pigs a suitable candidate for modelling a range of human diseases. Porcine lungs share several anatomical and histological features with human lungs, including similar tracheobronchial tree structure, and abundance of airway submucosal glands [90]. Pigs have previously been used to model pulmonary diseases involving inflammation and infection such as chronic bronchitis [91]. Furthermore, as human CF lung disease progresses over the lifetime of an individual, the longevity of pigs allows for long-term investigations of lung disease pathogenesis and assessment of therapeutics, which is not possible in rodent or ferret models due to their relatively short lifespan or disease severity, respectively [90]. The pig CFTR amino acid sequence is also 95% conserved with human, and the electrophysiological properties of porcine airway epithelium and submucosal glands resemble those observed in humans [36, 92].

Adeno-associated virus-mediated gene targeting of CFTR was used to generate the first CF pig models containing either a null allele (CFTR^−/−^) or the common Phe508del mutation (CFTR^ΔF508/ΔF508^) [6, 93]. Crossbreeding of CFTR^+/−^ and CFTR^+/ΔF508^ heterozygotes was also performed to produce a third model, denoted CFTR^-/ΔF508^ [94]. Shortly thereafter, Klymiuk et al. generated another knockout CF pig using an alternative approach that involved sequential targeting of CFTR using bacterial artificial chromosome (BAC) vectors [7]. Following breeding of the first CF pigs, it was realised that maintenance of the animals requires intensive and costly husbandry due to the severe gastrointestinal phenotype. Newborn CF pigs have a 100% penetrance of meconium ileus, with piglets requiring surgery soon after birth to relieve the obstruction. CF pigs also demonstrate pancreatic insufficiency and other gut-related issues requiring treatments including enzyme replacement therapy to aid digestion, oral vitamin supplements to prevent malnutrition, oral proton pump inhibitors or H2 blockers to control gastric acid, and laxatives to prevent bowel obstruction [93]. Using the same approach employed to create the gut-corrected CF mouse and ferret models, a transgenic CFTR^−/−^ pig was generated to express porcine CFTR in the intestines thereby overcoming the need for surgical correction of meconium ileus at birth [95].

#### Airway disease in CF pig models

CFTR^−/−^, CFTR^ΔF508/ΔF508^ and CFTR^-/ΔF508^ pig strains (hereafter collectively referred to as CF pigs) spontaneously develop key features of human CF lung disease within months of birth. These manifestations include airway inflammation, infection, tissue remodelling, mucus accumulation, and obstruction [94]. As in human CF populations, the severity of the lung phenotype varies between animals, with individual lobes also demonstrating heterogeneity [93]. Examination of neonatal CF pig airways shows no evidence of inflammation, comparable to newborn humans with CF [94, 96]. BAL profiles also indicate no difference in leukocyte and interleukin 8 (IL-8) concentrations between newborn CF and non-CF pigs. Over time however, inflammation ensues, ranging from mild to severe leukocytic infiltration, with severe cases occasionally exhibiting ulceration and abscess formation of the airway wall, and destruction of submucosal glands. Shortly after birth, CF pig lungs demonstrate defective bacteria eradication with newborn CF pigs challenged with *Staphylococcus aureus* failing to eradicate the bacteria as effectively as wild-type pigs [94]. This defective bacterial killing has been attributed to reduced airway surface pH in the CF pig and subsequent diminishing of the ASL antimicrobial function [78].

CF pigs demonstrate heterogeneous airway remodelling, with some cases showing evidence of goblet cell hyperplasia, airway wall thickening, and rarely, distended submucosal glands [94]. As observed clinically in infants with CF [97, 98], CF pigs present with airway obstruction comprising of atelectasis, hyperinflation, air trapping, and pneumonia [99]. Purulent material appears to obstruct the trachea and bronchi, often containing bacteria, neutrophils, and macrophages [94]. Tracheal abnormalities are also observed in CF pigs including a triangular rather than circular shaped trachea [7], smaller lumen area and circumference, irregular cartilage rings, and altered smooth muscle [93, 100]. Similar tracheal malformations have been noted in CF mouse models [43], CF rats [4], and infants with CF [100]. Interestingly, newborn CF pigs do not demonstrate reduced PCL depth in the trachea [101], but they do exhibit impaired MCT [102].

A range of ion transport measures performed on the nasal, tracheal and bronchial epithelia of CF pigs using tissues, cultures and in vivo approaches reveals electrophysiological defects consistent with loss of CFTR activity [101]. These abnormalities tend to be more pronounced in CFTR^−/−^ airway epithelia when compared to CFTR^ΔF508/ΔF508^ pigs, most likely due to the CFTR^ΔF508/ΔF508^ epithelia retaining some residual CFTR function [93]. Characteristic of CF, CFTR-mediated chloride transport is substantially reduced in the nasal, tracheal, and bronchial epithelium of CF pigs when compared to wild-type. CF pig nasal epithelia also exhibits a CF-like response to amiloride perfusion, but the trachea does not. Further investigation into this phenomenon revealed that the amiloride response observed in the CF pig nasal epithelia was due to reduced CFTR-mediated chloride conductance rather than sodium hyperabsorption [101]. Excised tracheal tissue from CF pigs also demonstrates diminished bicarbonate conductance, which is consistent with the human CF phenotype [93, 101].

Given that CF pigs capture many features of human CF airway disease, they have been useful for several research applications including investigations of lung disease pathogenesis [94], studying electrolyte transport defects [101], and exploring mechanisms involved in CFTR-Phe508del biosynthesis and misprocessing [92]. CF pigs have also provided a useful platform for exploring the origins of inflammation and infection within CF airways [94], identifying the role of ASL acidification in lung disease development [78], and trialling viral-mediated airway gene therapy approaches [103, 104]. In the future, CF pigs may also be useful for the long-term testing of therapeutics such as CFTR modulators, and for assessing airway disease prevention strategies [105].

### Concluding remarks

CF mouse models have proven useful for researching many facets of CF, but their use for airway-related investigations is restricted because they only exhibit mild features of CF lung disease, and induced airway disease using bacterial challenge approaches has limitations. Although the nasal epithelium of CF mice has provided a platform for exploring pathogenesis and assessing therapeutics, the lack of overt lower airway disease manifestations and the high degree of phenotype heterogeneity between strains means that new models are required. The β-ENaC mouse has provided a useful alternative to CF mice as it models lower airway obstruction, although it is not suitable for all applications (e.g. trialling CFTR-directed therapies) as CFTR expression and function are unaffected. Endeavours to overcome deficiencies of the available mouse models has led to development of new CF animal models including CF rat, ferret, and pig models. These models demonstrate an array of CF lung disease phenotypes with varying severity, as outlined in Table 1.

**Table 1:** Airway phenotypes of CF animal models compared to humans

	Human	CFTR knockout/mutant mice	β-ENaC mouse	Rat	Ferret	Pig
ASL HEIGHT IN LOWER AIRWAYS	Reduced [107]	NR in lower airways Reduced in nasal epithelium [15]	Reduced in bronchi [69, 70]	Reduced in trachea [4, 77, 80]	NR	Normal in trachea of newborn pigs [101]
MUCOCILIARY CLEARANCE IN LOWER AIRWAYS	Impaired [108]	Impaired in trachea of some strains [45] (has been contested [46])	Impaired in trachea [70]	Normal in young rats (< 3 months) [4] Impaired in the trachea of older rats (> 3 months) [77, 80]	Impaired in trachea [86]	Impaired in trachea [102]
REDUCED CHLORIDE SECRETION IN AIRWAYS	Present [109, 110]	Present in nasal epithelium [21] Absent in lower airways [25]	Absent [69]	Present in nasal and tracheal epithelium [4]	Present in tracheal epithelium [5, 87]	Present in nasal, tracheal and bronchial epithelium [93, 101]
SODIUM HYPERABSORPTION IN AIRWAYS	Present [48, 110–112] (has been contested [109])	Present in nasal epithelium [49–51] Absent in lower airways [25]	Present in trachea [69]	Absent in nasal and tracheal epithelium [4]	Absent in trachea [86, 87]	Absent in nasal and tracheal epithelium [101]
MUCUS OBSTRUCTION IN LOWER AIRWAYS	Mucus plugging [113]	Absent [15]	Mucus plugging [69]	Increased stored nasal mucus [4] and preliminary evidence of mucus plugging of submucosal glands in large airways [77, 80]	Mucus plugging [86]	Mucus plugging [93]
LUNG INFECTION	Present [114]	Absent [21]	Present in neonates but reduces with age [72]	Absent [4]	Present [86]	Present [94]
AIRWAY INFLAMMATION	Present [115]	Absent [21]	Present [69]	Absent [4]	Present [86]	Absent in newborn pigs but develops overtime [94]

*NR = not reported*

*Data insufficient for* CF *rabbit models*

Rats have intrinsic advantages for routine use as a model of CF including low cost and maintenance, rapid reproduction rate, and large litter sizes. The CFTR knockout rat demonstrates many pulmonary phenotypes including airway surface defects, a CF-like electrical profile in nasal and tracheal tissues, abnormal mucus production, and tracheal malformations. Although promising, further in-depth characterisation of the airway disease that is present, or readily inducible, would aid investigators in using this model for research applications.

The lung disease modelled by the CF ferret shares several similarities to that observed in humans. The concurrent presence of airway inflammation, infection, and mucus obstruction provides a highly valuable platform for further elucidating lung disease pathogenesis, as well as trialling therapies in a lung environment that closely replicates that of a human CF patient. However, the severe disease phenotype of the CF ferret model continues to limit its use, as the ferrets often have poor survival and require ongoing antibiotic treatment, therefore additional husbandry needs may constrain investigations.

Pigs share several airway characteristics with humans that make them suitable for developing a model of CF lung disease. Recently generated CFTR knockout and Phe508del mutant pig models exhibit lung disease hallmarks including inflammation, airway obstruction, and infection, traits that will facilitate both basic and pre-clinical airway-related research. However, CF pig models have drawbacks that may hinder their usability including the need for costly therapies to prolong survival of piglets, and the inherent costs and logistical limitations that arise when using large animal species for research.

Recently, CRISPR/Cas9 has been used to generate CFTR knockout and Phe508del mutant CF rabbit models [8]. Rabbits are considered to be an ideal species for modelling CF lung disease, as their airways are similar to humans in terms of anatomy and inflammatory responses [106]. Initial characterisation reports of the CF rabbit models reveal a CF-like lung phenotype including evidence of sodium hyperabsorption in trachea, mucus plugging, and bacterial infection in the lower airways, with BAL predominately containing bacteria from the *Streptococcus* and *Staphylococcus* genera [8]. Although these findings are preliminary, the CF rabbit models show promise for future use in CF-related research.

Animal models will continue to play a fundamental role in furthering our understanding of CF. Along with the development of rapid and precise gene editing technologies such as CRISPR/Cas9 comes the potential for new species to be used as platforms for modelling CF, as well as modification of existing models by introduction of human-specific CFTR mutations. Animal models that accurately recapitulate the hallmark features of human CF lung disease will be crucial in providing researchers with a resource for trialling experimental lung therapies, identifying new treatment targets, and elucidating the complex mechanisms that underlie CF lung disease. Although a perfect model of CF lung disease does not exist, each animal model has unique advantages and can be used in complementary manner to investigate CF-related questions.

## Abbreviations

ASL: Airway surface layer
BAL: Bronchoalveolar lavage
CACC: Calcium-activated chloride channel
CF: Cystic fibrosis
CFTR: Cystic fibrosis transmembrane conductance regulator
cAMP: Cyclic adenosine monophosphate
ENaC: Epithelial sodium channel
FABP: Fatty acid binding protein
I_sc_: Short circuit current
MCC: Mucociliary clearance
MCT: Mucociliary transport
NPD: Nasal potential difference
PCL: Periciliary liquid
siRNAs: Short-interfering RNAs
SPF: Specific pathogen free
